# Emotional remodeling with oxytocin durably rescues trauma-induced behavioral and neuro-morphological changes in rats: a promising treatment for PTSD

**DOI:** 10.1038/s41398-020-0714-0

**Published:** 2020-01-27

**Authors:** Claire Le Dorze, Antonella Borreca, Annabella Pignataro, Martine Ammassari-Teule, Pascale Gisquet-Verrier

**Affiliations:** 1 Université Paris-Saclay, CNRS, Institut des Neurosciences Paris-Saclay, 91190 Gif-sur-Yvette, France; 2 Santa Lucia Foundation, via del fosso di fiorano 64, 00143 Rome, Italy; 3 Institute of Translational Pharmacology, National Research Council, Rome, Italy

**Keywords:** Learning and memory, Psychiatric disorders

## Abstract

Recent evidence indicates that reactivated memories are malleable and can integrate new information upon their reactivation. We injected rats with oxytocin to investigate whether the delivery of a drug which dampens anxiety and fear before the reactivation of trauma memory decreases the emotional load of the original representation and durably alleviates PTSD-like symptoms. Rats exposed to the single prolonged stress (SPS) model of PTSD were classified 15 and 17 days later as either resilient or vulnerable to trauma on the basis of their anxiety and arousal scores. Following 2 other weeks, they received an intracerebral infusion of oxytocin (0.1 µg/1 µL) or saline 40 min before their trauma memory was reactivated by exposure to SPS reminders. PTSD-like symptoms and reactivity to PTSD-related cues were examined 3–14 days after oxytocin treatment. Results showed that vulnerable rats treated with saline exhibited a robust PTSD syndrome including increased anxiety and decreased arousal, as well as intense fear reactions to SPS sensory and contextual cues. Exposure to a combination of those cues resulted in c-fos hypo-activation and dendritic arbor retraction in prefrontal cortex and amygdala neurons, relative to resilient rats. Remarkably, 83% of vulnerable rats subjected to oxytocin-based emotional remodeling exhibited a resilient phenotype, and SPS-induced morphological alterations in prelimbic cortex and basolateral amygdala were eliminated. Our findings emphasize the translational potential of the present oxytocin-based emotional remodeling protocol which, when administered even long after the trauma, produces deep re-processing of traumatic memories and durable attenuation of the PTSD symptomatology.

## Introduction

Intrusions of traumatic memories and flashbacks, that occur either spontaneously or in response to trauma cues, are core symptoms of the post-traumatic stress disorder (PTSD). Hence, a variety of therapeutic protocols aimed at decreasing the emotional valence of traumatic cues (e.g., extinction or exposure therapies) have been proposed^1^ but their efficacy is questioned by regular observation of a high rate of relapse^2–5^.

We recently presented evidence indicating that when reactivated, memory becomes highly malleable so that any information delivered close to those phases is integrated with the former representation and can potentially modify its original content^6^. In line with this view, treatments known to reduce the emotional response before the reactivation of a trauma memory should lead to the formation of a new memory with a reduced emotional content that would not be expected to trigger PTSD symptoms. Supporting this view, we showed that a single amphetamine injection, administered in rats shortly before the reactivation of a remote trauma memory, was sufficient to reduce expression of PTSD-like symptoms monitored one month after treatment^7^. Considering the therapeutic potential of this process referred to as emotional remodeling, we wondered whether administration of amphetamine-like compounds with mood-stabilizing and anxiolytic/fear reduction properties might counteract even more durably PTSD symptoms and the neural changes they depend on. Among those, methylenedioxy methamphetamine (MDMA), also known as ‘ecstasy’, has been used with success as an adjunct for psychotherapy by a number of California therapists for treatment-resistant PTSD (see ref. ^8^) but was later discarded for potential risks of addiction. Interestingly, it has been proposed that anti-PTSD MDMA effects could be ascribed to a MDMA-induced release of oxytocin^9,10^. Oxytocin is a non-addictive nona-peptide, which is synthesized in the paraventricular and supraoptical nuclei of the hypothalamus of vertebrates and which exerts a wide spectrum of central and peripheral effects as neurohormone, neurotransmitter, or neuromodulator^11^. In the central nervous system, it is released by two neuronal populations of the paraventricular nucleus, the magnocellular neurons which project to the posterior pituitary, and the parvocellular neurons which project on the anterior pituitary, directly into the circulation^12–14^. The oxytocin effects are transduced via the oxytocin receptors abundantly expressed in regions involved in emotions and cognition like the hippocampus, the septum, and the amygdala^15,16^.

In rodents, intracerebral infusion of oxytocin reduces arousal in non-stress conditions^17^ and decreases reactivity to fear-associated cues^18^. In humans, oxytocin increases social approach by attenuating anxiety and stress, and globally contributes to promote “trusting behavior”^19,20^, thereby suggesting that it could be used to treat psychiatric disorders associated with a dysregulation of emotional control.

In humans, oxytocin delivered shortly after trauma^21,^^22^ was reported to prevent the manifestation of the PTSD symptomatology. Indeed, the temporal contiguity between trauma and treatment suggests an acute effect of oxytocin on the neural support of a partially stabilized memory. Differently, if a stable memory returns to be malleable upon its reactivation, the administration of oxytocin during the malleability phase might lead to the formation of a novel, less traumatic, memory no longer able to generate PTSD symptoms. To investigate this possibility, we first exposed rats to the single prolonged stress (SPS) paradigm of PTSD. Two weeks later, we characterized their profile of vulnerability or resilience to trauma on the basis of their anxiety and arousal scores^23^. Following another 2-week interval, rats were administered intracerebral infusions of oxytocin or saline before their exposure to trauma reminder cues. The therapeutic effect of the treatment was then evaluated following other 2 weeks by controlling whether the behavioral and neural alterations that were specific to the vulnerable phenotype were alleviated in oxytoxin-treated vulnerable rat.

## Methods

### Animals

The subjects were 50 male Sprague Dawley rats (Harlan Laboratories, France), housed in pairs with food and water available ad libitum, 250–275 g (7/8 weeks), at the beginning of the experiments. After a 2-week period, rats were regularly handled and weighed. All experiments were approved by the ethics committee CEE59 (ethics approval number 2015-07) in accordance with the European Communities Council Directive [2010/63/EU, 22 September 2010].

### Trauma induction

Trauma was induced by coupling training in an inhibitory avoidance task with SPS. Sensory cues delivered during SPS were subsequently used as trauma reminders^24,25^.

#### Inhibitory avoidance task

Inhibitory avoidance training took place in a plexiglas chamber (49 × 22 × 21 cm) divided into two equal size compartments (one white, one black) by a sliding door. The floor in the black compartment consisted of metal grids spaced 1.2 cm apart, and the grids were connected to a shock source. A 40 W bulb was suspended above the center of the white compartment. Each rat was placed in the white compartment (white safe box) for 1.5 min, then the light was turned on and the sliding door separating the two compartments was opened. Once the rat entered the black compartment, the sliding door was closed and two foot-shocks (0.5 s; 0.5 mA) were delivered separated from 5 s. The controls rats were placed in the same conditions but did not receive any shock. Immediately thereafter, rats were exposed to the SPS.

#### Single prolonged stress (SPS)

Rats were exposed to three different stressors. They were first restrained for 2 h in a transparent conical Plexiglas restriction container (21 × 7.5 cm) with a hole in the small end of the cone allowing them to breath. Immediately after, they were placed for 20 min for a forced-swim in a water tank (57.5 cm of depth; 26.5 cm of diameter) filled two-thirds from the bottom with water (26 ± 1 °C) in such a way they were unable to rest by using their tail for support. Upon removal from the water tank, they were gently dried and placed in a resting cage (45-cm long × 24-cm wide × 20-cm high) located under a lamp for 15 min. Finally, they were put in a small chamber (19.5 × 14 × 11.5 cm), saturated in CO_2_ induced by dry ice until loss of consciousness (about 45–50 s). They were then quickly removed, placed in the resting cage where they were observed until complete recovery, and then returned to their home cage. All steps were done individually. SPS was induced in the presence of a tone (metronome pulsations (120 bpm) present throughout SPS) and an odor (piece of cotton soaked with 5 µl of acetophenone (Sigma-Aldrich), placed in proximity to the restriction container). These cues were later used as trauma reminders. Control rats were exposed to the same cues and apparatuses as experimental rats: they were first placed in a cage next to the restraint tube during 2 h, then on a support (30-cm high, 26-cm diameter), placed in an empty water tank. After a 15-min rest in a cage under a lamp, they were enclosed in the CO_2_ box without dry ice.

### Vulnerability and resilience profiling

Two weeks after their exposure to SPS, rats were divided into resilient and vulnerable on the basis of their anxiety profile in the elevated plus maze (EPM) which estimates their propensity to explore open spaces, and of the intensity of their acoustic startle response (ASR), i.e., their reaction to a sudden, potentially threatening, acoustic stimulus.

Behavior scoring A flexible video tracking system (ANY-mazeTM, Stoelting Co, Wood Dale, USA) was used for automated scoring in the elevated plus maze, and to assess reactivity to trauma-associated cues tests. A video camera was placed above or next to the apparatus and the information was relayed to a monitor in an adjoining room to score the behavior in real time. Measures of freezing defined as the absence of all movement, except for respiratory, were made manually, during exposures to trauma-associated cues, with the help of an ethologic keyboard on ANY-maze. All behavioral tests were done with an experimenter blind to the group conditions. To ensure the reliability of freezing measurement, experimenters were first trained with previous ANY-maze videotapes until their analyses match with the former ones. In addition, the main experimenter regularly checked the measurement through random re-reading of experimental videotapes.

#### Elevated plus maze (EPM—day 15)

The maze consisted of four arms (50-cm long and 11-cm wide, two open and two closed with 50-cm high walls) made of black plastic material and arranged in a cross with two arms of the same type facing each other. Rats were individually placed at the arms intersection—facing a closed arm—and allowed to explore the maze for 5 min. Rat’s full body entering an open (12 lux) or closed arm (2 lux) was scored as an entry while an exit was defined as an entry in another arm. The percentage of time spent in the open arms (time in the open arms/(time open arms + closed arms) × 100), was used as the anxiety index^26,27^, with greater anxiety represented by a lower index. The session duration was 10 min.

#### Acoustic startle response (ASR—day 17)

Two startle chambers (Columbus Instruments, Ohio, USA) were used. Each chamber (60 × 45 × 43 cm) included a ventilated, sound-attenuated lighted box providing a 68 dB background noise and containing a Plexiglas box (29 × 16.5 × 25 cm) equipped with a speaker and fitted with a piezoelectric accelerometer. To restrict movements during the test, rats were confined in a wire mesh cage (20 × 13 × 8 cm) located within the Plexiglas box. The ASR session began with a 5-min period of acclimatization, followed by a series of five white noise sounds (1 dB; 20 ms; 20 s inter-sound-intervals) to define the baseline. Trials consisted of 30 consecutive presentations of 30 ms, 115 dB sounds (white noise), delivered over a 16-min period with a variable inter-sound interval (mean = 20 s). ASR scoring consisted in subtracting the baseline amplitude from the mean amplitude recorded during the 30 presentations. Because the rat body weight can act as a confounding factor (e.g., little startle can be scored as a high ASR in heavy rats), the rat body weight (in grams) was used as a corrector factor of ASR amplitude (in mV) according to the formula: mV × grams/100.

#### Criteria for determination of vulnerability or resilience to trauma

A rat was considered to show a PTSD-like symptom when it was scoring over half a standard deviation of the scores distribution of control rats. Rats exhibiting 2 symptoms were assigned to the vulnerable group while the remaining rats were assigned to the resilient group.

### Emotional remodeling protocol

#### Cannula implantation (day 21)

Rats were anesthetized with a mixture of ketamine (75 mg/kg) and xylazine (10 mg/kg) (0.2 ml/100 g body weight i.p) and placed in a stereotaxic frame (Kopf Instruments). Holes were drift in the skull at the AP (+1.0 mm) and ML (+1.6 mm), coordinates from bregma^28^. A guide cannula (21 G; rats, 12 mm length, Phymep, France) was lowered above the right lateral ventricle 1.8 mm ventral to bregma. The guide cannula was secured on the skull with screws, immersed in dental cement (Dentalon), and closed by a stainless steel dummy cannula. Correct placements of the cannula within the lateral ventricle was checked during brain slices collection.

#### Emotional remodeling sessions (days 29–31)

Rats received two remodeling sessions beginning with an oxytocin (Sigma-Aldrich, Saint Quentin Fallavier, France) or saline (0.9%) infusion delivered 40 min before the trauma reactivation induced by the exposure to a trauma-associated cue. Oxytocin was administered i.c.v. at a dose of 0.1 µg (in 1 µl of saline) chosen on the basis of previous studies^17,29,30^, as well as of preliminary experiments showing that it significantly reduced anxiety in traumatized rats after acute ICV injections.

The infusions was performed via an infusion cannula (25G, extended 2 mm beyond the guide cannula) connected via polyethylene tubing to a 10-µl syringe (Hamilton) during 10 min (1 µl/min) delivered in a new room dimly lighted (15 lux). On the first session (day 29), rats were placed in the white safe box of the inhibitory avoidance apparatus^22^. On the second session (day 31), they were placed in a novel box and exposed to the SPS-associated tone (metronome, 120 bpm). Upon completion of each session, rats remained for 1 h in the dimly light room before they returned to the colony room.

### Effect of oxytocin-based emotional remodeling on PTSD-like behavioral symptoms

**Fig. 1:**
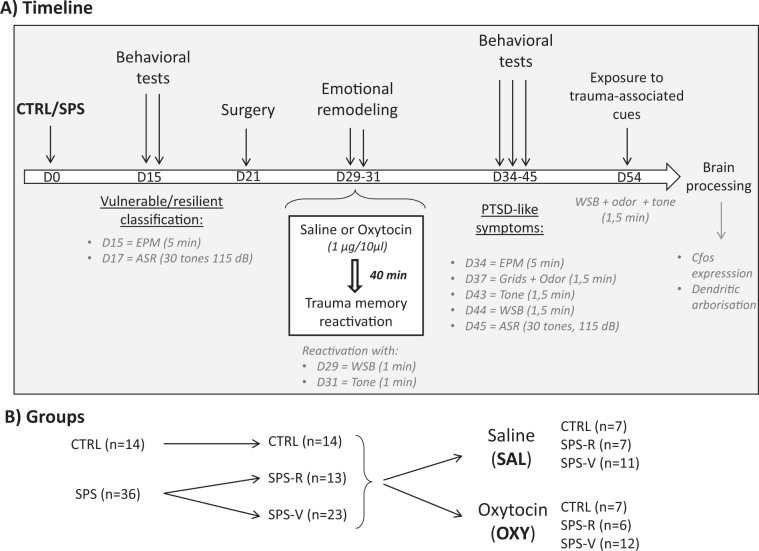
General method. **a** Timeline of the experimental design: Rats were first exposed to the trauma (inhibitory avoidance immediately followed by single prolonged stress (SPS)) or to a control (CTRL) situation. Two weeks later, they were classified as vulnerable or resilient on the basis of their anxiety scores in the EPM and of their ASR. The remodeling treatment was administered on days 29 and 31. From days 34 to 45, rats were exposed to several behavioral tests for an evaluation of PTSD-like symptoms. On day 54, rats were exposed to a combination of three trauma-associated cues and their brains were immediately processed for c-fos immunochemistry and morphological analyses. EPM: elevated plus maze; ASR: acoustic startle response; WSB: white safe box; tone/odor: exposure to the trauma-associated tone/odor. **b** Groups: number of rats in the control (CTRL) and traumatized (SPS) groups. SPS rats were then classified as resilient (R) and vulnerable (V), before receiving an emotional remodeling under saline (SAL) or oxytocin (OXY).

The efficacy of the emotional remodeling treatment was assessed in drug free conditions by controlling anxiety and arousal, and then exposing rats to SPS reminders over a period ranging from 3 (day 34) to 24 (day 54) from oxytocin infusions (see Fig. 1 for procedure details).

#### Exposure to the EPM (day 34)

Rats were placed in the EPM and their anxiety scores were recorded with the same method as on day 15.

#### Exposure to the grids plus odor (day 37)

Rats were placed for 90 s in a cage with grids similar to the grids delivering electrical shock in the inhibitory avoidance and containing the SPS-associated odor (a filter paper soaked with 5 µl of acetophenone placed above the grids), and their freezing responses were recorded.

#### Exposure to the SPS-associated tone (day 43)

Rats were placed in a cubic box and exposed to the SPS-associated tone (metronome 120 bpm) for 90 s, and their freezing responses were recorded.

#### Exposure to the white safe box (WSB—day 44)

Rats were exposed for 90 s to the safe white compartment of the inhibitory avoidance apparatus placed in the SPS room and their freezing responses were recorded.

#### Measurement of the ASR (day 45)

Rats were placed in the startle chambers and the amplitude of their ASR was measured as on day 17.

#### Exposure to a combination of 3 cues (day 54)

Rats were exposed to a combination of SPS-associated cues (odor and tone delivered in the safe compartment of the inhibitory avoidance chamber), and their freezing responses were recorded.

### Effect of oxytocin-based emotional remodeling on PTSD-associated neural symptoms

On day 54, rats were deeply anesthetized (lethal pentobarbital injection) 90 min after their last exposure to the combination of 3 SPS cues and their brain were removed. One hemisphere was processed for immunofluorescence detection of c-fos-labeled cells in the prelimbic (PL) region of the medial prefrontal cortex (mPFC) and in the basolateral amygdala (BLA). The other hemisphere was processed for Golgi–Cox impregnation to measure dendritic length extension and the number of nodes on dendritic trees in the same regions. Right and left hemispheres were randomly assigned to each measurement. Quantification was carried by experimenters blind to the experimental condition.

#### c-fos immunofluorescence labeling and quantification

One hemisphere was immersed for 4 h in a 4% PAF solution (paraformaldehyde in 0.1 M phosphate buffered saline) transferred in 30% saccharose, embedded in Tissue-Tek OCT (Sakura) and then cut coronally (40-µm thick) at −18 °C using a cryostat. Free-floating sections were stored in multi-well plates and immunofluorescence assays were carried out in 3–5 sections per region along their entire rostro-caudal extension. Sections were incubated overnight at 4 °C with the antibody against c-fos (1:200, Abcam Chip Grade Ab 7963) diluted 1:500 in phosphate buffer (PBS) containing 0.2% Triton X-100. On the following day, they were incubated for 2 h in the secondary antibody TRITC (tetramethylrhodamine isothiocyanate, diluted 1:200 in PBS containing 0.2% Triton X-100) antirabbit (Jackson ImmunoResearch). To co-visualize c-fos expression with cell nuclei, sections were counterstained with 4,6-diamidino-2-phenylindole (DAPI, 1 µg/ml for 3 min), mounted on polysined P glasses, and coverslipped with fluoromount. Images of c-fos immunostaining in the prelimbic (PL) region of the medial prefrontal cortex and in the basolateral nucleus of the amygdala (BLA) were acquired with a confocal microscope (Zeiss LSM700) at a ×20/zoom 1 magnification. In each region of interest (ROI), four sub-regions were randomly selected for automatic detection of c-fos immunoreactive spots by IMARIS software (version 7.6.5). For each ROI, the counts were obtained from the average of three representative coronal sections taken from the same rat. Counting of c-fos-labeled cells was made in the prelimbic (PL) region and the basolateral nucleus of the amygdala (BLA), by an observer blind to the experimental condition.

#### Dendrite arborization impregnation and quantification

The other hemisphere was immersed in a standard Golgi–Cox solution (1% potassium dichromate/1%mercuric chloride/0.8% potassium chromate) and stored at room temperature for 6 days: They were transferred to a sucrose solution (30%) for 5 days and then sectioned coronally (150 µm) using a vibratome. Sections were mounted on gelatinized slides, stained according to the Gibb and Kolb^31^ method, and covered with Permount. Layer II/3 PL neurons and spiny BLA neurons were identified under low magnification (×20/0.5NA) and subsequently analyzed under higher magnification (63X/0.75N) according to a previously used procedure^32,33^, In each experimental condition, five fully impregnated pyramidal neurons displaying dendritic trees without obvious truncations and isolated from neighboring impregnated neurons were retained for the analysis. Measurements were carried out using a microscope (DMLB, Leica) equipped with a motorized stage and a camera connected to a software for morphological analyses allowing quantitative 3D analysis of complete dendritic arborization (Neurolucida 7.5; MicroBrightField, Inc.). The length of the dendritic trees was quantified tracing the entire apical and basal dendrite arbor, and then performing Sholl analyses. Briefly, using the center of the soma as reference point, dendritic length, diameter, and branch points were measured as a function of their radial distance from the soma by adding up all values in each successive concentric segment (segment radius: 25 µm). Morphological measurements were made by an experimenter blind to the experimental conditions.

### Statistical analyses

ANOVAs were carried out using VAR3 software allowing post hoc planned comparisons^34^. Before treatment, between-group differences in behavior were assessed by means of one-way ANOVAs with group as the main factor. After treatments, between-group differences in behavior, c-fos-labeled cells, and dendrite length were assessed by means of two-way ANOVAs with group (CTRL, SPS-R, or SPS-V) and treatment (SAL or OXY) as main factors. The difference in the percentage of SPSV rats showing PTSD-like symptoms after OXY and SAL infusion was estimated by means of a chi-square test. Significant differences were set at *p* < 0.05.

## Results

### Vulnerability to trauma

**Fig. 2:**
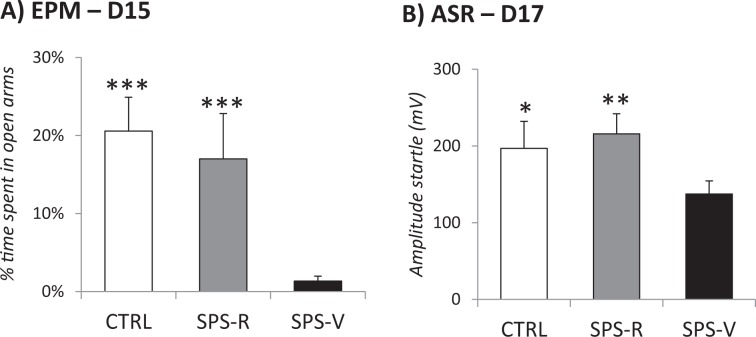
PTSD-like symptoms tests before the treatment. **a** EPM, performed at day 15: percentage of time spent in open arms, during a 5-min period. **b** ASR performed at day 17: mean startle amplitude (mV) obtained during the five blocks of six 115 dB tones. Data are expressed as mean ± standard error of the mean (SEM). *0.05 > *p* > 0.01; **0.01 > *p* > 0.001; ***p < 0.001.

A total of 50 rats was used in this study. Two weeks after the trauma (SPS), control (*n* = 14) and SPS (*n* = 36) rats were tested for anxiety (EPM) and arousal (ASR) to define vulnerable and resilient rats, according to previously validated criteria^16,35^. Vulnerable rats were those exhibiting EMP and ASR scores above ½ standard deviation of the CTRL group mean. Resilent rats were those scoring below that level. Among the 36 rats exposed to SPS, 23 rats (63.8%) exhibited PTSD-like symptoms and were defined as SPS-vulnerable (SPS-V), while the remaining 13 rats were considered as SPS-resilient (SPS-R). As illustrated in Fig. 2, this procedure allowed us to dissociate a resilient group similar to the control group from a vulnerable group showing performance that significantly differed from the two other groups. As expected, ANOVAs performed on these groups revealed significant group differences for anxiety on day 15 (F(2–48) = 14.44, *p* < 0.001) and for ASRs on day 17 (F(2–48) = 4.21, *p* = 0.019). Planned comparisons showed that SPS-V rats always differed from both SPS-R and CTRL rats with no difference between these two latter groups. As shown in Fig. 2, SPS-V rats spent significantly less time in the open arms of the EPM than both CTRL (F(1–35) = 38.40, *p* < 0.001) and SPS-R (F(1–34) = 19.68, *p* < 0.001) rats, and showed ASRs that significantly differed from both, CTRL (F(1–35) = 34.84, *p* < 0.001) and SPS-R rats (F(1–34) = 19.83, *p* < 0.001).

### Behavior

**Fig. 3:**
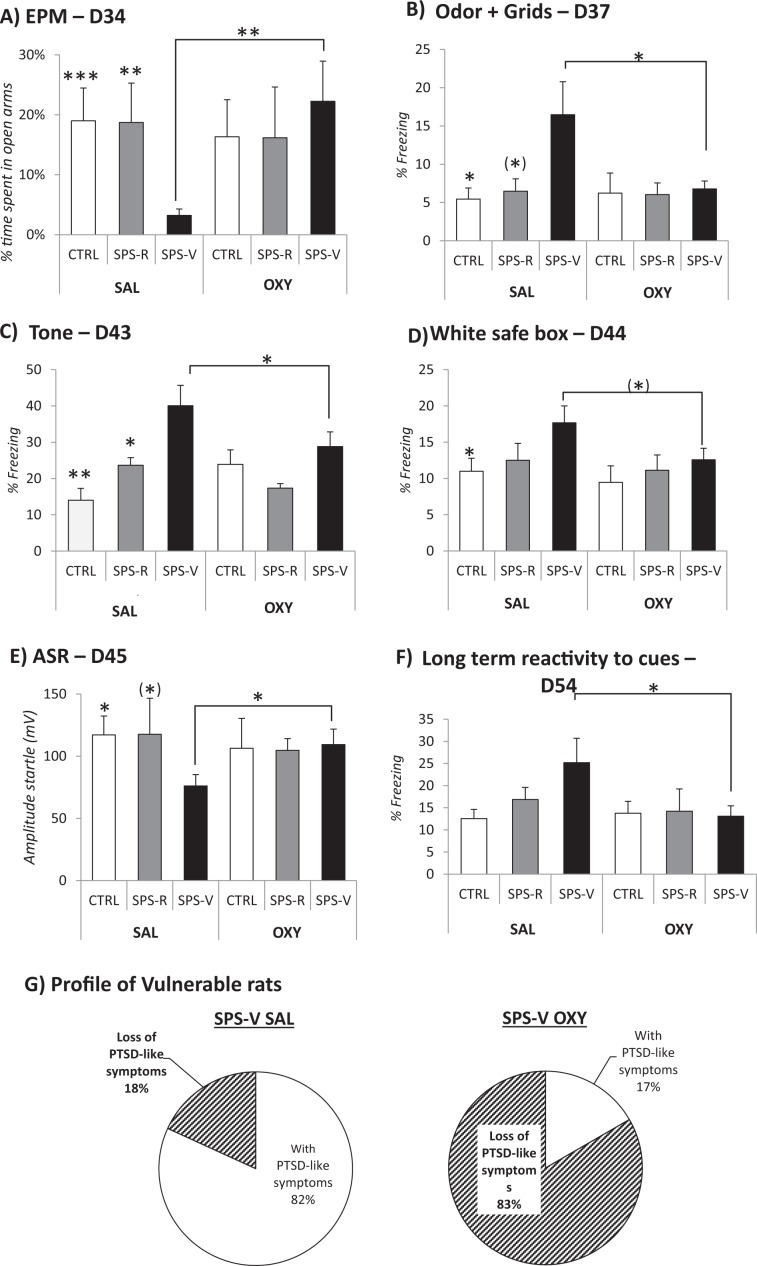
Effects of the remodeling treatment on PTSD-like symptoms in tests and reactivity to trauma-associated cues. Behavioral scores of control (CTRL) and traumatized (SPS-R and SPS-V) rats receiving a remodeling treatment with oxytocin (OXY) or saline (SAL). **a** Day 34: percentage of time spent in the open arms of the elevated plus maze (EPM), during a 5-min period of time. **b** Day 37: percentage of freezing shown in a box equipped with a grid and scented with the trauma-associated odor, during a 90-s period. **c** Day 43: percentage of freezing shown during the exposure to the trauma-associated tone, during a 90-s period. **d** Day 44: percentage of freezing shown in the white safe box of the avoidance apparatus, during a 90-s period. **e** Day 45: acoustic startle response (mV) obtained during the five blocks of six 115 dB tones. **f** Day 54: percentage of freezing shown during a 90-s exposure to the combination of three trauma-associated cues (tone, odor, and white safe box). **g** Profile of vulnerable traumatized rat after the remodeling treatment: percentage of rats showing vs those not showing the PTSD-like symptoms (OXY: oxytocin; SAL: saline). **a**–**f** Data are expressed as mean ± standard error (SEM). ^(^*^)^0.10 > *p* > 0.05; *0.05 > *p* > 0.01; **0.01 > *p* > 0.001; ***p < 0.001).

EPM (day 34, Fig. 3a): A significant Group effect in the SAL condition (F(2–22) = 6.52, *p* = 0.005) showed that vulnerable rats spent less time in the open arms than both control rats (F(1–16) = 15.04, *p* = 0.001) and resilient rats (F(1–16) = 10.51, *p* = 0.004).

Planned comparisons then revealed that oxytocin selectively increased the time spent in the open arms by vulnerable rats, but not in the other rats (treatment effect in the vulnerable condition, F(1–21) = 7.35, *p* = 0.011).

Grid and odor (day 37, Fig. 3b): A significant Group effect in the SAL condition (F(2–22) = 3.37, *p* = 0.05) showed that vulnerable rats exhibited stronger freezing upon exposure to the trauma odor cue (never exposed before), than both control (F(1–16) = 4.91, *p* = 0.05) and resilient rats (F(1–16) = 3.18, *p* = 0.09), although the latter effect did not reach statistical significance. Oxytocin decreased freezing in the vulnerable rats (treatment effect in the vulnerable condition, F(1–21) = 4.79, *p* = 0.038).

Tone (day 43, Fig. 3c): A significant Group effect in the SAL condition (F(2–22) = 7.76, *p* = 0.002) showed that vulnerable rats exhibited stronger freezing to the tone than both control (F(1–16) = 11.05, *p* = 0.004) and resilient rats (F(1–16) = 4.65, *p* = 0.043). Oxytocin decreased freezing in the vulnerable rats (treatment effect in the vulnerable condition, F(1–22) = 4.35, *p* = 0.05).

White safe box (day 44, Fig. 3d): A significant Group effect in the SAL condition F(2–22) = 3.05, *p* = 0.05) showed that vulnerable rats exhibited stronger freezing in the white safe box than control rats (F(1–16) = 4.4, *p* = 0.05). Oxytocin reduced freezing in the vulnerable rats (treatment effect in the vulnerable, F(1–22) = 3.28, *p* = 0.08) even though this comparison did not reach statistical significance.

Acoustic Startle response (day 45, Fig. 3e): A significant Group effect in the SAL condition (F(2–22) = 3.38, *p* = 0.05) showed that vulnerable rats exhibited lower acoustic startle responses than control rats (F(1–16) = 6.02, *p* = 0.02) and SPS-R rats (F(1–16) = 3.04, *p* = 0.09) even though this latter comparison did not reach statistical significance. Oxytocin increased startle responses in the vulnerable rats (treatment effect in the vulnerable condition, F(1–21) = 4.79, *p* = 0.038).

Long-term reactivity to a combination of 3 trauma-associated cues (day 55, Fig. 3f): Oxytocin reduced freezing in SPS-V rats during remote exposure to trauma cues (treatment effect in the vulnerable condition (F(1–21) = 4.64, *p* = 0.04).

### Post-remodeling vulnerability to trauma (days 34 and 45)

Late behavioral profiling carried out in vulnerable rats treated with oxytocin by scoring anxiety in the EPM (mean: 17.6% + 3.9%) and ASRs (mean: 101.4 + 12.5) showed that 10 out of 12 (83%) of them now behaved as resilient rats (Fig. 3g). In vulnerable rats treated with saline only 2 rats out of 11 (18%) exhibited a resilient profile (*X*² = 9.76, *p* < 0.001).

### C-fos expression

**Fig. 4:**
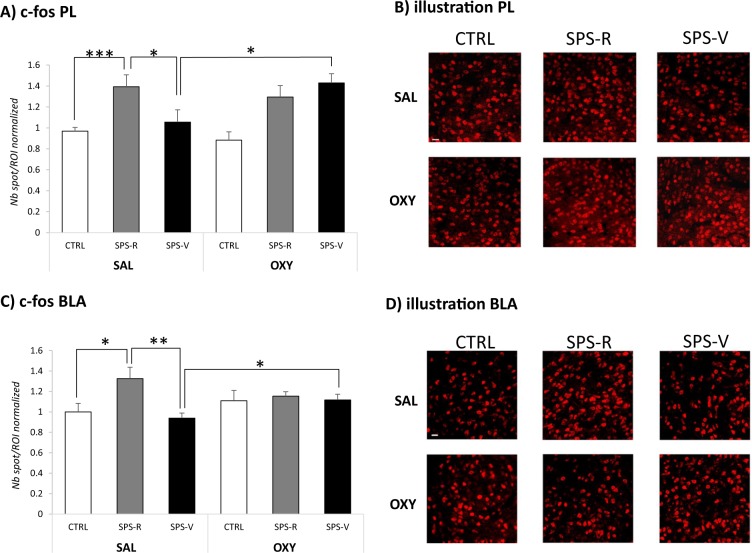
c-fos expression in the prelimbic cortex (PL) and the basolateral amygdala (BLA) of rats exposed to the combination of trauma-related cues 3 weeks after emotional remodeling treatment. **a** Number of c-fos spots/regions of interest (ROI) counted in the PL of rats in each experimental condition. **b** Representative images of c-fos immunoreactive puncta detected in the PL of control (CTRL), SPS-resilient (SPS-R), and SPS-vulnerable (SPS-V) rats injected with oxytocin or saline before reactivation of trauma. **c** Number of c-fos spots/ROI counted in the BLA of rats in each experimental condition. **d** Representative images of c-fos immunoreactive puncta detected in the BLA of CTRL, SPS-R, and SPS-V rats injected with oxytocin or saline before reactivation of trauma. Extension of sampling regions for c-fos counting in PL and BLA was 60 × 60 μm.

Statistical comparison of c-fos immunoreactive spots counted in the PL and the BLA revealed significant effects of Group (PL, F(2–66) = 8.78, *p* < 0.001; BLA, (F(2–57) = 5.31, *p* < 0.01) and Group × Treatment interactions (PL, F(2–66) = 4.31, *p* = 0.05; BLA, F(2–57) = 3.44, *p* = 0.037) (Fig. 4). In the SAL condition, c-fos expression was stronger in resilient rats than in control (PL, F(1–19) = 15.99, *p* < 0.001; BLA, F(1–16) = 5.69, *p* = 0.028) and vulnerable (PL, F(1–17) = 4.03, *p* = 0.05; BLA, F(1–14) = 10.01, *p* < 0.001) rats. In the OXY condition, c-fos expression did not differ between resilient and vulnerable rats due to the selective effect of oxytocin in the vulnerable condition (treatment effect, PL, F(1–24) = 7.32, *p* = 0.012; BLA, (F(1–20) = 4.46, *p* = 0.045).

### Dendritic arbor extension

**Fig. 5:**
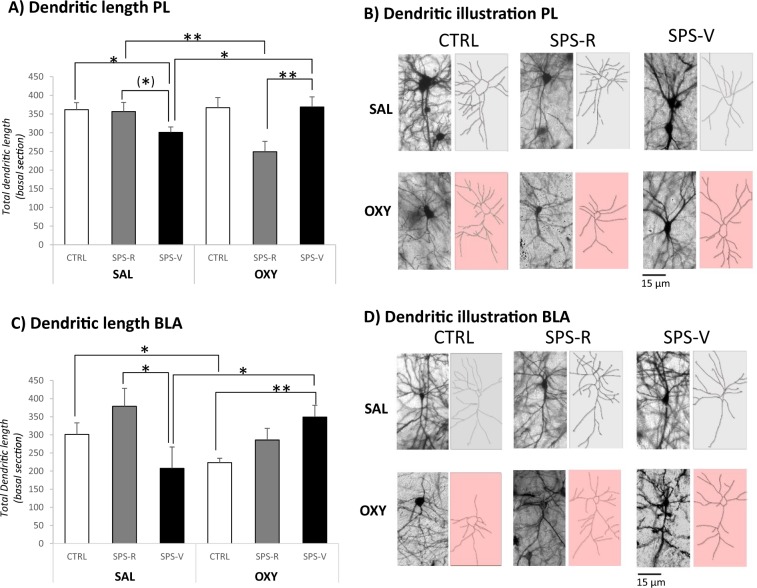
Dendritic arbor extension and complexity of prelimbic (PL) and basolateral amygdala (BLA) neurons of rats exposed to the combination of trauma-related cues 3 weeks after emotional remodeling treatment. **a** Total dendritic length (in µm) in the prelimbic cortex of rats in each experimental condition. **b** Representative images of the dendritic arbor extension detected in the PL of control (CTRL), SPS-resilient (SPS-R), and SPS-vulnerable (SPS-V) rats injected with oxytocin or saline before reactivation of trauma. **c** Total dendritic length (in µm) in BLA neurons of rats in each experimental condition. **d** Representative images of the dendritic arbor extension detected in the BLA of control (CTRL), SPS-resilient (SPS-R), and SPS-vulnerable (SPS-V) rats injected with oxytocin or saline before reactivation of trauma. Data are expressed as mean ± standard error (SEM). ^(^*^)^0.10 > *p* > 0.05; *0.05 > *p* > 0.01; **0.01 > *p* > 0.001; ***p < 0.001.

Statistical comparison of dendritic length measured from 10 to 60 µM from the soma revealed significant Group × Treatment interactions (PL: F(2–54) = 6.94, *p* = 0.002; BLA: F(2–54) = 5.60, *p* = 0.006) (Fig. 5).

In the SAL condition, vulnerable rats exhibited shorter dendrites in PL and BLA neurons when compared with resilient rats (PL: F(1–18) = 3.78, *p* = 0.06; BLA: F(1–18) = 4.93, *p* = 0.037). In the OXY condition, the treatment exerted an opposite effect depending on the phenotype to which it was administered. In particular, oxytocin increased dendritic length in the vulnerable condition (treatment effect, PL: F(1–18) = 4.84, *p* = 0.03; BLA: F(1–18) = 4.39, *p* = 0.048) while it decreased dendritic length in the resilient (PL: F(1–18) = 8.33, *p* = 0.009) or control conditions (BLA F(1–18) = 5.14, *p* = 0.03).

## Discussion

Our results show that three weeks following emotional remodeling with oxytocin, 83% of the rats identified as vulnerable to trauma became resilient, indicating the durable efficacy of our treatment. Remarkably, the behavioral and neural characteristics of vulnerable rats treated with oxytocin entirely aligned with those of control and resilient rats treated with saline.

### PTSD-like behavioral symptoms

SPS-vulnerable rats exhibited enhanced anxiety and increased arousal consistent with our previous observations in rats undergoing the same SPS procedure^24,35^. Regarding fear symptoms, vulnerable rats showed intense freezing when exposed to SPS-related cues during a period extending from 29 to 54 days post-trauma. The robustness and persistence of these symptoms confirm the validity of SPS as a PTSD model^36,37^.

### Emotional remodeling with oxytocin and behavior

Oxytocin-based emotional remodeling reversed the manifestation of PTSD-like behavioral symptoms in rats that were characterized as vulnerable to trauma. Specifically, oxytocin decreased anxiety in the elevated plus maze test, a result already noticed in several studies^18,21^. Oxytocin also increased arousal and reduced hyper-reactivity to trauma cues, as repeatedly reported in alcohol dependent rats and humans^38^. In those reports, however, the alleviation of anxiety and hyper-reactivity to trauma cues was observed when the subjects were under the influence of oxytocin. Differently, here we show that the association of oxytocin with trauma-cue exposure reverses the manifestation of those symptoms even weeks after treatment cessation. Interestingly, the reduction of PTSD symptoms by oxytocin has been ascribed to a restoration of a normal pattern of functional connectivity between the amygdala, the insular cortex, the caudate nucleus and the anterior cingulate cortex^39^, i.e., brain regions that we found to be activated upon the presentation of retrieval cues^40^. Of note, more than 80% of the vulnerable rats treated with oxytocin performed as did resilient rats while only 5% of vulnerable rats treated saline showed a resilient-like performance. Remarkably, oxytocin rescued the vulnerable behavioral profile with no relapse being observed over a 3-week period. Importantly for potential translational applications, oxytocin had no behavioral effect in control and resilient rats which rules out the likelihood of undesirable side effects. Such a high percentage of complete and durable remission following oxytocin treatment reveals the therapeutic potential of anxiolytic/mood-stabilizing drugs administered in phases of memory malleability.

### Emotional remodeling with oxytocin and c-fos expression

Exposure to reminder cues of aversive/traumatic situations reliably altered c-fos expression in stress circuitry regions^41–43^. Unexpectedly, when exposed to SPS-associated cues, resilient rats, which behaved as did control rats, were those showing the highest levels of c-fos expression in both the PL and the BLA. This observation is not entirely new, as co-activation of the two regions supporting resilient copying has already been reported in PTSD models^42,44,45^, one explanation being that mPFC activation improves behavioral control^46^ in situations where potential dangers are signaled via amygdala activation^47^. Intriguingly, vulnerable rats exhibited less BLA and PL activation compared to resilient rats. Indeed, hypo-activation of the mPFC has been extensively reported in PTSD patients and animal models^48–52^, whereas hypo-activation of the BLA is very discrepant with the main stream of the literature^53^. However, protocols ranging from script-driven imagery in PTSD patients^54^ or exposure to cat odor^41^ or SPS in rats^43^, have shown that the amygdala is not systematically activated upon re-exposure of vulnerable subjects to trauma-related cues. Second, consistent with our findings, a decrease in c-fos expression has been detected in the BLA of vulnerable rats exposed to the traumatic experience with reminders of stress (TERS) model, which, as with our protocol, includes multiple exposures to stress cues^44^. Habituation and/or extinction resulting from repeated testing of anxiety, arousal, and fear reaction to SPS cues might attenuate PL and BLA activation without preventing the expression of fear which could be mediated by other nodes in the fear circuitry. This possibility is supported by the recent identification of novel neural substrates in the regulation of fear memory expression^55^. It is worth noting that while in our conditions, vulnerable rats were not able to activate the BLA and the PL in response to trauma reminders, emotional remodeling with oxytocin, which had no effect on control and resilient rats, not only reversed PTSD behavioral symptoms but also rendered the pattern of BLA and PL activation of vulnerable rats similar to the one of saline-injected resilient rats. This latter result suggests that the treatment rescues the mechanism at the origin of resilience; that is, the propensity to activate brain regions which mediate active coping with stress.

### Emotional remodeling with oxytocin and dendritic arbor complexity

The morphology of cortico-limbic neurons responds differentially to variations in duration and intensity of stressors as well as to the amount of time that elapsed since the stress. A majority of data shows that chronic stress elicits dendritic retraction in mPFC neurons and dendritic extension in BLA neurons^56^, while, acute stress produces dendritic retraction in both regions^57^. This latter pattern corresponds to morphological alterations found in SPS-vulnerable rats treated with saline. Remarkably, in line with the report that oxytocin increases neurite length and expression of cytoskeletal proteins associated with neuronal growth^58^, SPS-vulnerable rats treated with oxytocin showed a resilient dendritic phenotype. Of note, oxytocin produced a paradoxical dendritic retraction in neurons from control and resilient rats which did not impact behavior. Thus, although the explanation is currently unknown, it could be that the resilient and vulnerable profiles are associated with distinct neurite molecular properties differently modulated by oxytocin. Nevertheless, the opposite relationship between dendrite retraction and resilience to stress strongly suggests that oxytocin might re-establish a pattern of neuronal connectivity similar to the one of saline-injected resilient rats, an effect in line with the report that oxytocin normalizes amygdala functional connectivity in PTSD patients^59^.

### Emotional remodeling with oxytocin: what mechanisms?

Altogether, our findings show that pre-reactivation oxytocin treatment globally rescues the vulnerable phenotype. Among the mechanisms which have been proposed to explain the oxytocin therapeutic effects (see ref. ^5^), a first possibility is that oxytocin blocks the process of memory reconsolidation, considered to be required to re-stabilize a reactivated memory^60^. However, this hypothesis is unlikely for two main reasons. First, oxytocin cannot be considered as a reconsolidation blocker since disruption, facilitation or no effect on retention have all been reported^61,62^. Second, oxytocin does not solely suppress fear symptoms of vulnerable rats but rescues trauma-induced neural alterations, a result which cannot be expected from reconsolidation blockers. Another possibility is that oxytocin accelerated extinction of reactivity to trauma-associated, as the result of their repeated presentations. Although oxytocin has been shown to facilitate next day extinction of threat memory in humans^63^, its role in long-term stabilization of extinction implicitly suggests that it should mediate exceptionally robust extinction process to prevent the reinstatement of fear after remote exposure to the entire set of retrieval cues. Indeed, oxytocin possesses intrinsic pharmacological properties, which are suitable to tackle maladaptive fear. In humans, it decreases anxiety^19,64^ and induces a positive emotional state via neuroendocrine regulation of stress responses^65,66^. In rats, it decreases anxiety^17,65,67,68^ and attenuates conditioned fear by inhibiting, via its receptors in the central amygdala, the excitatory flow from the amygdala to brainstem sites that mediates fear responses^15,69^. In view of these properties, and of the general agreement that memory reactivation opens a malleability window during which any newly presented information can be integrated within the original memory representation^70,71^, we propose that pre-reactivation oxytocin treatment leads to the formation of a novel, less traumatic, fear memory representation which mediates resilience to trauma.

This hypothesis is in good agreement with the positive effects obtained by injecting the anxiolytic drug propranolol in cocaine addicted^72^ or PTSD^73^ patients before their respective exposure to drug-craving stimuli or to PTSD cues. It is also consistent with the evaluation of the recent advancements in MDMA-assisted psychotherapy for PTSD^74,75^ which points out that treatments that block pathological activation in brain regions implicated in the expression of fear during the memory malleability phase are likely to induce re-processing of traumatic memories. This new memory with a lower emotional component could therefore reduce the forthcoming impact of the trauma. Of note, PTSD therapies based on the induction of a positive state during memory reactivation (e.g., EMDR: movement desensitization, CBC: cognitive behavioral therapy) also exploit the malleability of reactivated memories^5^. Thus, EMDR or CBC protocols combined with oxytocin treatment should considerably enhance their therapeutic efficacy.

## Conclusion

In conclusion, oxytocin-based emotional remodeling, a treatment where oxytocin is delivered in association with memory reactivation, is effective in reversing the manifestation of behavioral and neural PTSD symptoms even long after its administration. It therefore appears as a promising therapy for durably tackling abnormally persisting memories with a high motivational/emotional content^5,7^.
